# Acetylation and deacetylation of histone in adipocyte differentiation and the potential significance in cancer

**DOI:** 10.1016/j.tranon.2023.101815

**Published:** 2023-11-05

**Authors:** Xiaorui Wang, Na Li, Minying Zheng, Yongjun Yu, Shiwu Zhang

**Affiliations:** a Department of Pathology, Tianjin Union Medical Center, Nankai University, Tianjin 300121, China; b Graduate School, Tianjin Medical University, Tianjin 300070, China

**Keywords:** Acetylation modifications, Adipocyte differentiation, Cancer stem cells, Polyploid giant tumor cells, Histones

## Abstract

Adipocytes are derived from pluripotent mesenchymal stem cells and can develop into several cell types including adipocytes, myocytes, chondrocytes, and osteocytes. Adipocyte differentiation is regulated by a variety of transcription factors and signaling pathways. Various epigenetic factors, particularly histone modifications, play key roles in adipocyte differentiation and have indispensable functions in altering chromatin conformation. Histone acetylases and deacetylases participate in the regulation of protein acetylation, mediate transcriptional and post-translational modifications, and directly acetylate or deacetylate various transcription factors and regulatory proteins. The adipocyte differentiation of stem cells plays a key role in various metabolic diseases. Cancer stem cells(CSCs) play an important function in cancer metastasis, recurrence, and drug resistance, and have the characteristics of stem cells. They are expressed in various cell lineages, including adipocytes. Recent studies have shown that cancer stem cells that undergo epithelial-mesenchymal transformation can undergo adipocytic differentiation, thereby reducing the degree of malignancy. This opens up new possibilities for cancer treatment. This review summarizes the regulation of acetylation during adipocyte differentiation, involving the functions of histone acetylating and deacetylating enzymes as well as non-histone acetylation modifications. Mechanistic studies on adipogenesis and acetylation during the differentiation of cancer cells into a benign cell phenotype may help identify new targets for cancer treatment.

## Highlights

• Adipocyte differentiation is regulated by many pathways, among which histone acetylation plays a key role as one of the important epigenetic modifications that can positively or negatively regulate adipocyte differentiation.
• Cancer stem cells and cancer cells undergoing epithelial mesenchymal transformation can be induced to become adipocytes thereby reducing malignancy.
• In the future, the research on the mechanism of acetylation regulation during adipocyte differentiation of cancer cells has the potential to be applied to cancer therapy.

## Graphical abstract

**Figure:**
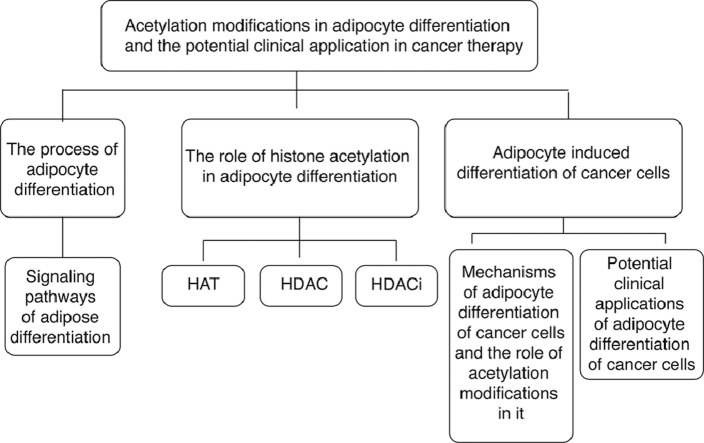

## Abbreviations

MCE, Mitotic clonal expansion; HATs, Histone acetyltransferases; HDACs, Histone deacetylases; MSCs, Mesenchymal stem cells; CSCs, Cancer stem cells; EMT, Epithelial-mesenchymal transition; PPARγ, Peroxisome proliferator-activated receptor γ; C/EBPs, CCAAT enhancer-binding proteins; aP2, Adiponectin 2; GLUT4, Glucose transporter 4; LPL, Lipoprotein lipase; WAT, White adipose tissue; BAT, Brown adipose tissue; Myf5, Myogenic factor 5; PGC-1α, PPAR-γ coactivator 1α; FOXO, Forkhead box protein O; SRC, Steroid receptor coactivator; PRDM16, PRD1-BF-1-RIZ1 homologous domain containing protein-16; RUNX1, runt-related transcription factor 1; TIF2, Transcription intermediary factor 2; GNAT, PCAF/Gcn-5-associated N-acetyltransferases; Gcn5, General control non-derepressible 5; PCAF, p300–CBP-associated factor; MYST, MOZ, Ybf2/Sas3, Sas2, and Tip60; MOZ, Monocytic leukemia zinc finger protein; Sas, Something about silencing; Tip60, Tat-interactive protein 60; USP7, Ubiquitin-specific protease-7; HBO1, Histone acetyltransferase bound to ORC1; ATF5, Activating transcription factor; MLL4, Mixed-lineage leukemia 4; BRD4, Bromodomain-containing protein 4; BET, Bromodomain and extra-terminal structural domain; Pol II, RNA polymerase II; SIRT, Sirtuin; NCoR, Nuclear Receptor Co-Repressor; RAR, Retinoic acid receptor; CoA, Coenzyme A; SAHA, Suberoylanilide Hydroxamic Acid; TSA, trichostatin A

## Introduction

Adipocyte differentiation is a complex physiological process that is divided into two main parts: differentiation of pluripotent mesenchymal stem cells (MSCs) into preadipocytes, and differentiation of preadipocytes into mature adipocytes through mitotic clonal expansion (MCE). Adipocyte differentiation is regulated by various transcription factors and signaling pathways [1,2]. Epigenetic modifications are heritable changes in gene expression that allow specific regions of the DNA to attract or repel proteins that activate genes, thereby activating or repressing their expression of specific genes in different cell types. Epigenetic inheritance occurs without altering the DNA sequence and can be stabilized during an organism's development and cell proliferation. Major epigenetic modifications include histone modification, DNA methylation, and RNAs [3,4].

Among these mechanisms, histone acetylation plays a crucial role in altering the chromatin conformation. Histone acetylation plays an important role in the regulation of gene expression. Histones are proteins that bind to DNA in the nucleus and belong to a family of basic proteins of five main types: H1, H2A, H2B, H3, and H4 [5]. The primary function of histones is the formation of octameric complexes that package DNA into structural units called nucleosomes. Acetylation of lysine in histone tails is highly dynamic and important for regulating chromatin structure, transcription, and DNA repair. Histone modifications exert their effects on specific enzymes, and the active sites of the enzymes are specific, with different enzymes modifying different sites or having different functions. Two competing families of enzymes, histone acetyltransferases (HATs) and histone deacetylases (HDACs), regulate histone acetylation [6]. Histone acetylation occurs mostly at the N-terminal lysine residues of histones H3 and H4. Histones are positively charged, whereas DNA is negatively charged, resulting in tight binding of histones to DNA. Histone acetylases transfer the acetyl group of acetyl coenzyme A to the lysine residues of histones, thereby neutralizing the positive charge of histones, weakening the interaction between DNA and histones, and relaxing the structure of the nucleosome so that DNA binds more readily to transcription factors; thus, histone acetylation is often associated with transcriptional activation. In contrast, HDAC deacetylate histones, which bind tightly to negatively charged DNA, densely coil chromatin, and repress gene transcription [7]. In addition to the acetylation of histones, non-histone proteins such as p53 can also be modified by acetylation. These modifications also play important roles in adipocyte differentiation [8].

The main cell sources for in vitro studies on lipogenic differentiation are MSCs, which can differentiate into multiple cell types such as C3H10T1/2 MSCs, and infinite cell lines that differentiate in a single adipose direction, such as 3T3-L1 preadipocytes [9,10]. Differentiation-induced therapies using cancer stem cells have been extensively studied in recent years. Cancer stem cells (CSCs) have MSC-like properties and can be induced to differentiate into benign phenotypes, such as adipose tissue, bone, and cartilage, which are sensitive to chemotherapeutic agents [11]. Additionally, cancer cells exhibit stemness following epithelial-mesenchymal transition (EMT) [12]. Cancer cells can be induced to differentiate into adipocytes by culturing them with inducing drugs. Daughter cells (PDC) of the polyploid cancer giant cell PGCC have been shown to have stemness and can be induced to differentiate into adipocytes [138]. PPARγ is the major transcription factor in adipose differentiation, and histone deacetylase can regulate the expression of acetylated PPARγ and p53 in adipose-differentiated HEY and MDA-MB-231 PDCs. In MDA-MB-231 PDCs harboring mutant p53, adipocyte differentiation was reduced by inhibiting the activity of histone acetylase p300, which suppresses the acetylation of p53 [13].

In this review, we outline the regulatory role of acetylation modifications in adipocyte differentiation, including the functions of histone acetylases and deacetylases, as well as histone deacetylase inhibitors, which have been extensively studied in recent years, as well as the functions of epigenetic readers. Understanding the role of acetylation in adipocyte differentiation will help to identify new regulatory sites and therapeutic approaches for the treatment of adipose tissue-related metabolic diseases and obesity. In particular, as the therapeutic modalities involving adipocyte differentiation in CSCs are better understood, the regulation of adipocyte differentiation-associated acetylation may play an important role in the clinical management of cancer.

### Classification of adipocytes and differentiation of progenitor cells

**Fig. 1:**
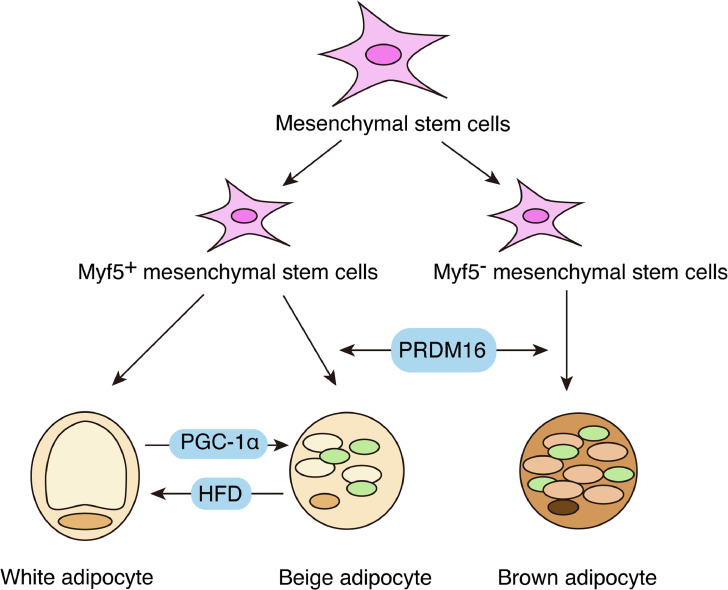
Differentiation of white, brown, and beige adipocytes. White adipocyte tissue (WAT) is derived from Myf5^+^ precursor cells, whereas brown adipocyte tissue (BAT) is generally derived from Myf5^−^ precursor cells. Beige adipocytes are homologous to white adipocytes and can also be differentiated from white adipocytes. WAT has large single-chambered lipid droplets and its main function is to store energy glycerides. Brown adipocytes in BAT are characterized by multi-chambered lipid droplets and mitochondria and dissipate energy in the form of heat through adaptive thermogenesis. Beige adipocytes have the same features as BAT.

There are three main types of adipocytes: brown, white, and beige. These are the functionally and morphologically distinct adipocyte groups. White adipose tissue (WAT) contains large single-chambered lipid droplets whose main function is to store energy glycerides in single-chambered white adipocytes. Brown adipocytes in the brown adipose tissue (BAT) are characterized by multichambered lipid droplets and mitochondria that dissipate energy in the form of heat through adaptive thermogenesis. Heat production by BAT is based on the action of uncoupling protein (UCP)−1 in the inner mitochondrial membrane, which solubilizes the mitochondrial proton gradient in the inner mitochondrial membrane, thereby uncoupling respiration from ATP [14]. In addition to WAT and BAT, beige adipocytes are adipocytes located within the WAT, with BAT characteristics, and which can be induced by cold stress [15]. Like BAT, beige fat burns and generates calories, both of which control energy homeostasis and regulate fat accumulation in the body [16]. There are differences in the origin of these three cell types, with WAT originating from myogenic factor 5^+^ (Myf5^+^) precursor cells and BAT generally originating from Myf5^−^ precursor cells. Overexpression of PPAR-γ co-activator 1α (PGC-1α) can cause browning of white adipocytes [17]. Beige adipocytes are similar to white adipocytes and can be differentiated from them. Irisin produced by muscle exercise stimulates the conversion of white fat cells into beige fat cells [16,18]. The ability of progenitor cells to differentiate into mature adipocytes depends on three levels of transcription factors and cofactors: epigenetic factors and microRNAs (miRNAs). C/EBPα acts in concert with PPARγ to establish the adipogenic phenotype [19]. The transcription factors PPARα and forkhead box protein O2 (FOXO2) and the PPARγ co-activators PGC-1α, steroid receptor co-activator-1 (SRC-1), and PRD1-BF-1-RIZ1 domain-containing 16 (PRDM16) promote brown adipogenesis. Transcription factors such as receptor-interacting protein 140 (RIP140) and transcription intermediary factor 2 (TIF2) promote WAT production [20], [21], [22] (Fig. 1).

### The process of adipocyte differentiation

**Fig. 2:**
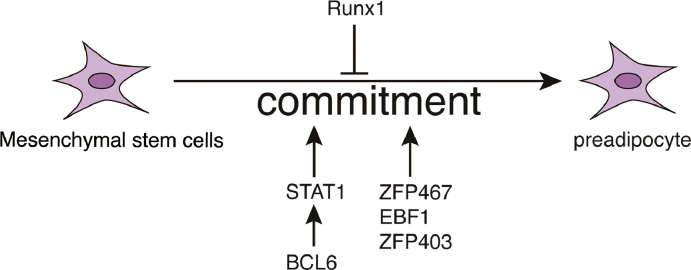
Adipocyte differentiation in pluripotent stem cells occurs in two stages: formation of precursor adipocytes and maturation of adipocytes. The first stage is called assay, in which pluripotent stem cells are transformed into preadipocytes that cannot be morphologically distinguished from pluripotent stem cells but lose the potential to differentiate into other cell types. The transcription factors Zfp 423, Zfp 467, EBF 1, and BCL 6 promote the stereotyping of preadipocytes into the adipogenic lineage, whereas RUNX 1 T1 inhibits this process.

Adipocyte differentiation in pluripotent stem cells occurs in two stages: the formation of precursor adipocytes and the maturation of adipocytes. The first stage is called commitment, in which pluripotent stem cells are transformed into preadipocytes that cannot be morphologically distinguished from pluripotent stem cells, but lose the potential to differentiate into other cell types. The transcription factors signal transducer and activator of transcription (STAT) 1, Zfp 423, Zfp 467, EBF 1, and B-cell lymphoma (Bcl) 6 promote the stereotyping of preadipocytes into the adipogenic lineage. Hu, et al. reported that mRNA expression of STAT 1 was increased and RNAi-mediated knockdown of STAT 1 inhibited the adipogenic stereotyping and differentiation in C3H10T1/2 cells. Bcl 6 can trans-activated STAT1 by directing binding to the 2031 and 1228 bp regions of the mouse STAT1 promoter [23]. Acetylation modification of STAT1 may negatively regulate the transcriptional activity of STAT1. HDAC inhibitors (HDACi) can inhibit the JAK-STAT and NFκB pathway by up-regulating the acetylation of STAT1 [24]. In HDACi or interferon α-stimulated melanoma cells, STAT1 was modified by acetylation by HAT CREB-binding protein (CBP) [25]. The acetylation of STAT1 also determined the sensitivity of ovarian cancer cells to cisplatin. Ovarian cancer cells treated with HDACi can promote the acetylation of STAT1 and reduce the drug resistance [26]. Runt-related transcription factor 1 (RUNX1) could inhibit the stereotyping of preadipocytes to adipogenic lineage [27]. By commitment, adipose stem cells are induced to form precursor adipocytes expressing the adipogenic transcription factors CCAAT enhancer binding protein (C/EBP) β and C/EBPδ (Fig. 2).

**Fig. 3:**
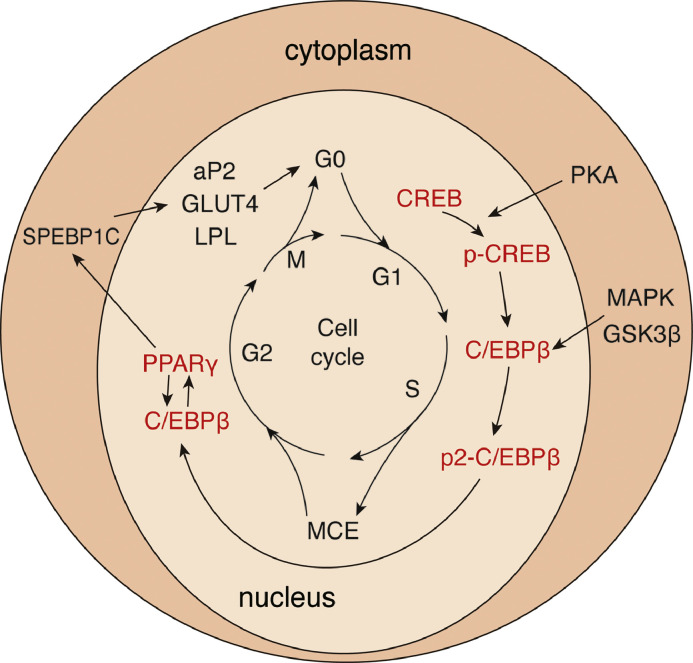
During the second phase of adipose differentiation, preadipocytes undergo growth arrest, MCE, early differentiation, and terminal differentiation eventually expressing various genes of mature adipocytes. Protein kinase A (PKA) catalyzes the phosphorylation and activation of CREB, and CREB expression activates C/EBPβ. C/EBPβ is hyperphosphorylated during the transition from G1 to S phase of the cell cycle, leading to the activation of glycogen synthase kinase-3β (GSK-3β) and mitogen-activated protein kinase (MAPK). c/EBPβ and C/EBPγ, among other C/EBPβ and C/EBPγ induce PPARy expression, and C/EBPα begins to synergize with PPARγ-activated lipid-associated factor expression.

In the second phase, preadipocytes gradually possess characteristics of mature adipocytes and acquire physiological functions, including lipid transport and synthesis, insulin sensitivity, and secretion of adipocyte-specific proteins. Expression of PPARγ and C/EBPα is central to preadipocyte stereotyping and terminal differentiation. The second phase was subdivided into four stages: growth arrest, MCE, early differentiation, and terminal differentiation [28]. Precursor adipocytes are cultured in cell culture plates, and after cell growth fusion reaches contact inhibition, growth slows down and gradually arrests, staying in the G0/G1 phase of the cell cycle. Proliferation arrest of precursor adipocytes is necessary to initiate precursor adipocyte differentiation. After growth arrest, the preadipocyte receives the appropriate mitotic and adipogenic signals and re-enters the cell cycle synchronously, undergoing several rounds of cell division, a process known as mitotic clonal expansion, which results in the loosening of the DNA structure to allow transcription factors to access the transcriptionally-regulated binding regions of specific genes in order to initiate the subsequent adipocyte differentiation program, a process dependent on the action of differentiation-inducing factors. C/EBPβ is hyperphosphorylated during the transition from the G1 to S phase of the cell cycle, leading to the activation of glycogen synthase kinase-3β (GSK-3β) and mitogen-activated protein kinase (MAPK). Blockade of C/EBPβ signaling prevents mitotic clonal expansion and adipocyte differentiation. Subsequently, transcription factors including C/EBPβ and C/EBPγ induce PPARy expression, and C/EBPα begins to synergize with the expression of lipid-associated factors activated by PPARγ [16]. PPARγ activates adipocyte protein 2 (aP2), phosphoenolpyruvate carboxykinase (PEPCK), glucose transporter protein 4 (GLUT4), lipoprotein lipase (LPL), and many other adipogenesis-related genes are transcribed [29]. It also activates the expression of C/EBPα, which in turn promotes the expression of PPARy, thus creating a positive feedback loop. At this stage, the morphology of adipocytes changes from spindle to spherical owing to the reorganization of the extracellular matrix and lysosomal proteins. When a cell enters the terminal differentiation stage, the ab initio synthesis of fatty acids increases significantly, and transcription factors and adipocyte-associated genes act synergistically to maintain the differentiation of precursor adipocytes into mature adipocytes. Adipocyte maturation is characterized by the ability of cells to synthesize and secrete a large number of protein and non-protein factors, some of which are involved in the endocrine regulation of energy homeostasis. PPARγ and C/EBPα are key regulators of adipogenesis, initiating and controlling the process of precursor adipocyte differentiation into mature adipocytes [30,31]. The increased expression of lipogenesis-related genes causes the deposition of triglycerides in adipose precursor cells, mediating their transformation into adipocytes, thus completing lipogenesis [32,33] (Fig. 3).

### Signaling pathway of HATs and HDACs involved in adipogenesis

#### MAPK/PPARγ signaling pathway regulates adipogenesis

The MAPK signaling pathway is important for regulating cell proliferation and differentiation and is mainly divided into extracellular signal-regulated kinases (ERKs), c-Junamino N-terminal kinases (JNKs), and p38 MAPK. ERK and p38 MAPK are involved in adipose differentiation [34,35]. ERK promotes early stages of adipogenesis. The upstream kinase MEK phosphorylates ERK, which can bind to the cAMP-response element-binding protein (CREB) and promote CREB phosphorylation [36]. The phosphorylated CREB interacts with HAT CREB-binding protein/EIA-associated 300-kDa proteins (CBP/p300) and binds to the proximal promoter of the *C/EBP* gene in 3T3-L1 preadipocytes, promoting C/EBPβ and C/EBPδ expression and participating in the early differentiation of adipogenesis [37]. Blocking ERK activity in 3T3-L1 cells and ESC can inhibit adipogenesis [38,39]. In terminal differentiation, ERK activation phosphorylates PPARγ, thereby inhibiting adipocyte differentiation [40]. P38 MAPK plays an active role in early fat differentiation. Specific inhibition of p38 MAPK blocks fat production in 3T3-L1 cells. Treatment with p38 inhibitor reduced C/EBPβ phosphorylation and PPARγ expression in vivo [41]. SIRT5 overexpression significantly downregulated ERK and p38 MAPK expression and inhibited phosphorylation of the MAPK pathway. Additionally, SIRT5 interference significantly upregulates the phosphorylation of the MAPK pathway, indicating that SIRT5 inhibits preadipocyte differentiation by suppressing this pathway [42].

#### The AMPK signaling pathway negatively regulates adipogenesis

Adenosine 5′-monophosphate (AMP)-activated protein kinase (AMPK) is an important regulatory enzyme that regulates cell metabolism and is involved in regulating bioenergy metabolism and maintaining cellular energy homeostasis [43]. The AMPK pathway plays an inhibitory role in regulating PPARγ and C/EBPα expression [44]. When AMPK is activated, it inactivates subtarget ACCs to attenuate adipogenesis by inhibiting the expression of FABP 4, FAS, and SREBP-1c. Overexpression of SIRT5 significantly up-regulated the phosphorylation level of the AMPK pathway and down-regulated the expression levels of PPARy, FXR, ACCa, and SCD 1, key genes in the AMPK pathway during preadipocyte differentiation, and inhibited preadipocyte differentiation and lipid synthesis through activation of the AMPK pathway [42]. The HDACi trichostatin A [TSA] can activate the AMPK signaling pathway as well, thereby inhibiting adipogenesis [45].

#### WNT signaling pathway negatively promotes adipogenesis

The WNT signaling pathway is associated with cell proliferation and differentiation. WNT proteins bind to coiled-coil receptors and initiate signaling through β-linked protein-dependent and non-dependent pathways [46,47]. WNT signaling inhibits the expression of PPARγ and C/EBPα, thereby playing an inhibitory role in adipocyte differentiation in vitro [48]. Some WNT protein inhibitors prevent the activation of typical WNT signals, including secreted frizzled-related proteins (sFRP), Dickkopf (Dkk), and WNT inhibitory factor (WIF) [49]. sFRPs are extracellular Wnt signaling antagonists that directly bind Wnt molecules. They sequester Wnts from their membrane-bound receptors and act as intracellular mediators of Wnt signaling by interacting with Dishevelled proteins, thereby inhibiting signaling from the Fz/LRP receptor complex. HAT SIRT1 is localized to the promoter of sFRP 2 and directly contributes to the aberrant epigenetic silencing of breast cancer cells. Overexpression of SIRT1 leads to the dephosphorylation of DACT and activation of the Wnt signaling pathway, and SIRT 1 may inhibit adipogenesis in MSCs by suppressing the expression of the Wnt signaling antagonists, sFRP 2 and Dapper homolog (DACT) 1 [50].

#### The RB signaling pathway is associated with adipogenesis

In the retinoblastoma protein (RB) signaling pathway, the RB-encoded pRb protein combines with E2F to form a complex. E2F in cells can combine with RNA polymerase to initiate gene transcription, and its combination with pRb inhibits the activity of E2F, thereby inhibiting the cell cycle [51]. RB promotes adipocyte differentiation by inducing cell cycle arrest and trans-activation of C/EBPs and recruits HDAC3 to the PPARγ target gene, forming the PPARγ-RB-HDAC3 complex, thereby inhibiting PPARγ and subsequently inhibiting adipose differentiation. After RB phosphorylation, the PPARγ-RB-HDAC3 complex can dissociate, thereby promoting adipogenesis [52].

### Acetylation modification of histones

**Fig. 4:**
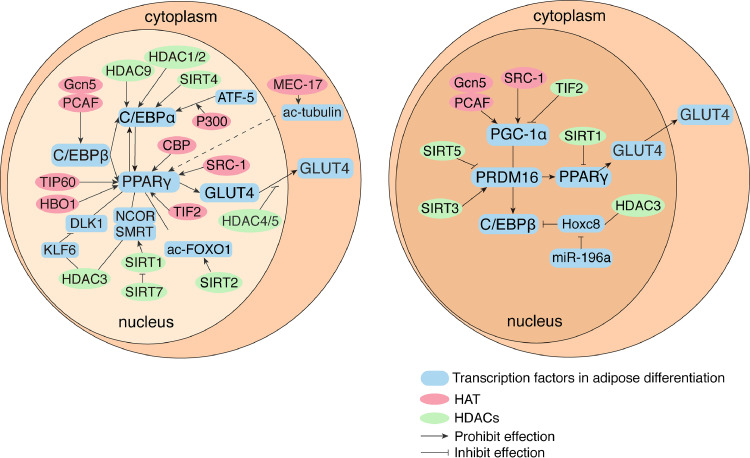
Histone acetyltransferases (HATs) and histone deacetylases (HADC) regulate transcription factors in adipocyte differentiation. A. Mechanisms of acetylase regulation in white adipocyte differentiation. PPARγ and C/EBPα are key regulators of adipogenesis that first initiate and monitor the entire lipogenic differentiation process. B. Mechanisms of acetylase regulation in brown adipocyte differentiation. PRDM16 is a key regulator of brown adipogenesis, promoting brown adipocyte production.

MSC differentiation is a complex process regulated by various signaling pathways with complex crosstalk involving epigenetic and transcriptional regulation [53,54]. Although previous studies have highlighted the regulation of MSC differentiation by external stimuli and internal transcription factors, recent studies have revealed the role of histone modifications in this process [55]. Transcriptional modifications of histone-modifying enzymes and histone tail fragments modulate gene expression, strongly influence MSC fate commitment, and are fundamentally involved in epigenetic regulation of adipogenesis [56]. Histone-modifying enzymes dynamically regulate the chromatin structure and gene expression by adding or removing large amounts of permissive or repressive histone marks. Typically, multiple lysine residues in histones 3 and 4 are acetylated during transcription. During this process, HATs catalyze the transfer of acetyl groups from acetyl coenzyme A to lysine residues. This leads to the neutralization of positive charges on histones, weakening their interactions with DNA [57,58]. Consequently, condensed DNA (heterochromatin) loosens and becomes transcriptionally hypersensitive in its open form (euchromatin) upon contact with DNA-binding transcription factors and RNA polymerase II [59,60]. Conversely, acetyl groups can be removed from acetylated histones by HDACs, at which point the nucleosomes become compact and DNA is no longer accessible, causing transcriptional repression and gene silencing [61]. Many histone-modifying enzymes and chromatin remodeling factors regulate the expression of different adipogenic genes during adipocyte differentiation [62,63] (Fig. 4).

### The role of histone acetyltransferases in adipocyte differentiation

#### Classification **of histone acetyltransferases**

HATs are classified into types A and B according to their cellular origins [64,65]. Type A HATs are usually associated with an increased expression of transcriptional activators and target genes. They are localized in the nucleus, where they transfer the acetyl group from acetyl-CoA to the e-NH2 motif of the histone N-tail after assembly into nucleosomes. Type A HATs are grouped according to their conserved core structures that are responsible for the recognition and binding of A (CoA). Based on their homology with yeast proteins, they are divided into three groups: PCAF/GCN-5-associated N-acetyltransferases (GNAT), monocytic leukemia zinc finger protein (MOZ), YBF2/something about silencing (SAS) 3, SAS2, and the tat-interactive protein 60 (TIP60/MYST) superfamily, which includes MOZ, YBF2, SAS2, TIP 60, and CBP/p300. Type B HATs are found in the cytoplasm where they acetylate newly formed histones, supporting their transport from the cytoplasm to the nucleus [66]. In type A HAT, PCAF/GCN-5-associated N-acetyltransferase (GNAT), and the MYST superfamily are usually acetylating H3H4 histones, whereas CBP/p300 is involved in the acetylation of H2A and H2B histones in addition to H3H4 histones. They interact with chromatin assembly factor-1 (CAF-1) complex to promote nucleosome alignment during DNA replication and repair [67]. The roles of different histone acetyltransferases in adipogenesis are listed in Table 1.

**Table 1:** The roles of histone acetyltransferases in adipogenesis.

Modifier	Target	Effect on adipogenesis
GGC5/PCAF	H3K9	Promote adipogenesis by acetylating the C/EBP-β promoter region [70]
TIP60 HBO1	H3K9	An adipogenic transcriptional co-factor that promotes adipogenesis [74] Interacts with FAD24 to promote adipogenesis by controlling DNA replication [76]
CBP/p300	H3K18/H3K27	Promote adipogenesis and contribute to PPARγ activation [77]
SRC1/2/3		Promote adipogenesis [82]
TIF2		Interacts with SARC1 and promotes adipogenesis [85]
MEC17		Promotes adipogenesis by acting on microtubule acetylation [88]

#### The GNAT family promotes adipocyte differentiation by facilitating C/EBPβ transcription

GNAT belongs to a family of homologous histone acetyltransferases important for normal development, cell proliferation, and differentiation. GNAT generally uses acyl-CoA to acetylate its substrates and plays a key role in regulating the myogenic program and adipocyte proliferation [68]. General control non-derepressible 5 (GCN5) and p300–CBP-associated factor (PCAF) are members of the GNAT acetyltransferase family that redundantly regulate histone H3K9 [69]. GCN5 and PCAF regulate the acetylation of the C/EBP-β promoter region during NIH3T3 and 3T3-L1 preadipocyte differentiation, have a suppressive effect on the cell cycle, prolong transcription, and control the expression upstream of PPARβ. GCN5 and PCAF are indispensable catalytic factors in adipogenesis, and their dual deletion inhibits PPARγ, resulting in severe defects in adipogenesis; however, these defects can be repaired by the ectopic expression of PPARγ [70]. GCN5 and PCAF also mediate the acetylation of H3K9 or transcription factors and regulate the recruitment of polymerase II (Pol II) to regulate the expression of PRDM16, a major regulator of brown adipocyte-specific features [71]. In addition to acetylating histones, PCAF can also act as an acetyltransferase in the acetylation of various transcription factors, such as P53, drosophila mothers against decapentaplegic acid (SMAD), E2F transcription factor 1 (E2F1), and NOTCH. PCAF and p300/CBP form acetyltransferase complexes that act as co-activators or acetylate histones and non-histones to activate transcriptional regulation [72].

#### The MYST family plays a positive role in the mitotic clonal expansion of adipogenesis

MYST is named after its founding members, monocytic leukemia zinc finger protein (MOZ), Ybf2/something about silencing 3 (SAS3), SAS2, and tat-interactive protein 60 (TIP60). TIP60 and histone acetyltransferase bound to ORC1 (HBO1) play important roles in adipogenesis [73]. TIP60 acetylates histones and non-histones, and is essential for adipocyte differentiation. TIP60 interacts with PPARγ, and its recruitment is critically dependent on PPARγ. The endogenous TIP60 protein is recruited to the PPARγ-target genes *FABP4* and perilipin in mature 3T3-L1 adipocytes, but not in preadipocytes. siRNA-mediated reduction of TIP60 impairs differentiation of 3T3-L1 preadipocytes. These findings demonstrate that TIP60 is an adipogenic transcriptional co-factor. During the first stage of 3T3-L1 differentiation, TIP60 protein expression increases, suggesting that the regulation of TIP60 protein levels plays an important role in early adipogenesis [74]. Ubiquitin-specific protease 7 (USP7)-mediated deubiquitination regulates TIP60 protein levels, and knockdown of TIP60 or USP7 inhibits mitotic clonal amplification during the basic step of adipocyte differentiation [75]. Similar to TIP60, *HBO1*-knockdown impairs the ability of 3T3-L1 cells to differentiate into mature adipocytes by inhibiting mitotic clonal amplification. Additionally, adipocyte differentiation factor 24 (FAD24) interacts with HBO1 to promote adipogenesis by regulating DNA replication [76].

#### CBP/p300 positively regulates adipocyte differentiation by promoting C/EBPα and PPARγ transcription

CBP and p300 are responsible for the acetylation of H3K18 and H3K27 in mammalian cells. The HAT activities of CBP and p300 are required for adipocyte differentiation and maintenance of mature adipocyte function [37]. CBP and p300 are important for the activation of PPARγ, and their downregulation significantly reduces adipocyte differentiation. In the early differentiation of the adipogenic process, CBP/p300 is involved in cell proliferation, development, and differentiation, as a co-regulator of C/EBP mediator subunit 1 and the gene encoding the master regulator of adipocyte differentiation, PPARγ2 [77]. Downregulation of CBP or p300 expression alone in 3T3-L1 preadipocytes corresponds to a reduction in PPARγ target gene expression and inhibition of adipocyte differentiation, suggesting that although they share a high degree of sequence similarity, they are not fully complementary in the regulation of adipocyte differentiation, indicating that the two enzymes may act at different points in adipogenesis [78]. Activating transcription factor 5 (ATF5) enhances C/EBPα transcriptional activation through direct interaction with C/EBPα, by binding to the C/EBPα promoter. p300 plays an important role in the interaction of ATF5 with C/EBPβ and acetylates ATF5 to enhance this process [79].

BRD4 is a bromodomain-containing protein and is a member of the bromodomain and extra-terminal structural domain (BET) family of proteins. BRD4 binds to acetylated histones and transcription factors and acts as an epigenetic reader involved in reading acetylated lysine residues. As an epigenetic reader, it binds to active enhancers and promoters to control the induction of cell identity genes [80]. Brd4 binds to active enhancers during adipogenesis and preferentially binds to active promoters after adipogenesis. Deficiency of Brd4 in preadipocytes inhibits PPARγ expression and prevents adipogenesis and adipogenic induction. CBP/p300 cooperates with H3K4 methyltransferase mixed-lineage leukemia 4 (MLL4) and Linage-400 deterministic transcription factor (LDTF) to recruit BRD4 to activate enhancers. MLL4 promotes chromatin opening during adipocyte differentiation, while CBP promotes enhancer activation after MLL4 initiates enhancers [81]. This promotes the binding of multiple transcriptional regulators (mediators, transcription initiation factor [TFIID], Pol II, positive transcriptional elongation factor b [p-TEFb], and eRNA) to the promoters of adipogenesis-related genes.

#### Nuclear receptor co-activators cooperate with PPARγ to promote transcription

Nuclear receptor co-activators can function as co-activators of PPARγ in a ligand-independent manner, similar to transcriptional co-activators. SRC and transcriptional intermediary factor 2 (TIF-2) are important nuclear receptor coactivators that display HAT activity. The HAT structural domain of SRC-1 is located in the C-terminal region and interacts with CBP/p300 and PCAF. Co-expression of SRC-1 and PPARγ enhances the transcriptional activity and adipogenesis of this factor [82]. p/CIP is a nuclear receptor co-activator. The deletion of p/CIP and SRC-1 inhibits BAT development and the expression of several PPARγ target genes in BAT. SRC-2 and SRC-3 promote the early differentiation of adipogenesis in humans [83,84]. TIF-2 is another nuclear receptor co-activator with HAT activity that interacts with CBP/p300. TIF2-deficiency in mice reduced PPARγ activity in WAT and decreased fat accumulation [85]. Astaxanthin, an oxygenated carotenoid, increases the interaction of PPARγ with TIF2 and SRC-1 and reduces the interaction of PPARγ with CBP [86].

#### MEC17 enhances adipocyte differentiation by promoting microtubule acetylation

MEC17 is another acetyltransferase conserved from *Tetrahymena* to mammals [87]. Cytoskeletal remodeling, including the disruption of microtubules (MT), a major component of the cytoskeleton, is a limiting step in the morphological transformation during adipogenesis, and the disruption of MT leads to an increase in intracellular triacylglycerol accumulation and significantly promotes adipogenesis. The expression of acetylation-resistant microtubule protein mutants significantly inhibits adipogenesis, indicating that adipocyte differentiation depends on microtubule protein acetylation. In contrast, MEC-17 directly promotes microtubule acetylation and acts synergistically with the deacetylases SIRT2 and HDAC6 to promote adipocyte differentiation [88].

### The role of histone deacetylase in adipocyte differentiation

HDACs deacetylate histone lysine residues, remodel chromatin, and play important roles in gene transcription [89]. The 18 known mammalian HDACs are divided into four classes based on their functions and sequence homology. Classes I, II, and IV HDACs are zinc-dependent, whereas class III HDACs are NAD+-dependent. Class I HDACs (HDACs 1, 2, 3, and 8) are widely expressed in human cell lines and nucleolar tissues. Class II HDACs (4, 5, 6, 7, 9, and 10) can translocate into the nucleus and respond to certain cellular signals they receive, exhibit tissue-specific expression, and shuttle between the nucleus and cytoplasm. Class III HDACs, also known as sirtuins (SIRT1–7), have a catalytic mechanism different from that of other HDACs. Class IV includes only one member, HDAC11, which is localized in the nucleus, but in activity assays, HDAC11 co-precipitates with cytoplasmically localized HDAC6 [90,91]. Similar to HATs, HDACs have several non-histone substrates, such as p53, heat shock protein 90 (Hsp90), transcription factor T cell factor (TCF), and β-catenin. HDACs deacetylate nucleosomes and deacetylate histone proteins, which bind tightly to negatively charged DNA; chromatin is densely coiled and is involved in adipocyte differentiation and the maintenance of the preadipocyte phenotype by repressing the transcription of key genes [92]. The downregulation of HDAC expression and the resulting increase in histone acetylation play essential roles in promoting adipogenesis. Only when the cellular levels of HDAC fall below a certain threshold can preadipocytes initiate differentiation. The roles of histone deacetylases in adipogenesis are listed in Table 2.

**Table 2:** The role of histone deacetylases in adipogenesis.

Class	Subtype	Subcellular location	Effect on adipogenesis
Ⅰ	HDAC1/2	Nucleus/nucleus and cytoplasm	Inhibits adipogenesis by repressing C/EBPα, regulates adipocyte differentiation in a redundant manner [93]
	HDAC3	Nucleus and cytoplasm	Promotes adipogenesis [101]
Ⅱa	HDAC4/5	Nucleus and cytoplasm	Promotes adipogenesis by regulating the *GLUT4* promoter [104]
	HDAC9	Nucleus	Inhibits adipogenic differentiation through an adenosine acetylase-independent mechanism [106]
Ⅲ	SIRT1	Nucleus and cytoplasm	Promotes or inhibits adipogenesis at different stages of adipogenic differentiation [115,116]
	SIRT2	Cytoplasm and nucleus	Promotes FOXO1 binding to PPARγ and subsequently inhibits PPARγ transcriptional activity to suppress adipogenesis [123]
	SIRT3	Mitochondrial matrix and cytoplasm	Promotes adipogenesis [124]
	SIRT4	Mitochondrial matrix	Promotes adipogenesis[128]
	SIRT5	Mitochondrion, cytoplasm, and nucleus	Promotes brown adipogenesis in bovine preadipocytes by activating AMPK and inhibits MAPK during bovine preadipocyte differentiation [42]
	SIRT6	Nucleus and endoplasmic cytoplasm	Inhibits adipogenesis and acts synergistically with SIRT5 [42]
	SIRT7	Nucleus and cytoplasm	Promotes adipogenesis by inhibiting the autocatalytic activation of SIRT1 [133]
Ⅳ	HDAC11	Nucleus	Inhibits adipogenesis [108]

#### HDACs function as inhibitors or promoters at different stages of adipocyte differentiation

HDAC1 and HDAC2 are homologous proteins that inhibit adipogenesis [93]. During this process, HDAC1 is recruited to the C/EBPα promoter in the early differentiation of adipogenesis to repress its transcription and interact with the pH domain-containing protein, casein kinase 2-interacting protein 1 (CKIP-1) [92]. Knockdown of HDAC1 and HDAC2 effectively inhibited adipogenesis induced by intracellular lipid accumulation in mouse embryonic fibroblasts, whereas deletion of HDAC1 or HDAC2 alone did not have the same effect. This suggests that HDAC1 and HDAC2 regulate adipocyte differentiation in a redundant manner [94]. In contrast, in glucocorticoid receptor-mediated preadipocyte differentiation, HDAC1 plays a major down-regulatory role by reducing C/EBPα and PPARγ expression during differentiation [95]. The co-blocking complexes of mSin3A and HDAC1 preferentially interact with C/EBPβ and reduce its potential for transcriptional activation, whereas glucocorticoids promote adipocyte differentiation by inducing degradation of this complex [96].

HDAC3 can interact directly with the transcriptional molecules nuclear receptor co-repressor (NCOR) and silencing mediator for retinoic acid and thyroid hormone receptor (SMRT) to form a stable ternary complex. Alternatively, HDAC3 can engage in transcriptional repression by binding to retinoblastoma protein (RB) and participating in the regulation of the adipocyte phenotype during early differentiation [97], [98], [99]. RB recruits HDAC3 to PPAR target genes and attenuates PPAR-mediated adipocyte differentiation. Phosphorylation of RB or inhibition of HDAC activity may disrupt the PPAR-RB-HDAC3 complex, thereby stimulating adipocyte differentiation [52]. HDAC3 also interacts with Kruppel-like factor 6 (KLF6) to inhibit *DLK1*, a gene encoding an epidermal growth factor-like homotrimeric transmembrane protein that is downregulated during adipocyte differentiation [100]. In a study using mouse liver cells, inactivation of HDAC3 was found to affect adipogenesis, and treatment of these mice with a PPARγ antagonist partially reversed lipid accumulation in the liver, suggesting that PPARγ contributes to an increase in lipid levels following HDAC3 inactivation [101].

The HOX genes are developmental genes that play important roles in cell differentiation. One of these, homeobox C8 (*HOXC8*), is expressed more in WAT than in BAT and is classified as a white adipose gene that directly represses the expression of C/EBPβ, a major regulator of brown adipogenesis, of which miR-196a is an upstream regulator of *HOXC8*. HOXC8 recruits HDAC3 and regulates C/EBPβ in concert with HDAC3 [102]. During adipocyte differentiation, recruitment of HDAC1 and HDAC3 to the endogenous *LPL* promoter is regulated by the cell cycle protein D1 [103]. HDAC4 and HDAC5 specifically regulate the promoter of the glucose transporter *GLUT4* to inhibit adipocyte differentiation. They are functionally redundant with the *GLUT4* promoter in preadipocytes; however, HDAC5 binds preferentially to the *GLUT4* promoter in preadipocytes. HDAC4 binds to *GLUT4* only in the preadipocyte stage when HDAC5 is inactivated. Therefore, despite its structural similarities, HDAC4 does not compete with HDAC5 for the regulation of the *GLUT4* promoter in preadipocytes [104].

HDAC9 inhibits adipogenic differentiation via a non-adenosine acetylase-dependent mechanism and HDAC9 overexpression inhibits adipogenesis in 3T3-L1–381 cells. Preadipocytes isolated from *HDAC9-*knockout mice show accelerated adipogenesis. In preadipocytes, *HDAC9* is transcribed by upstream stimulatory factor 1 (USF1) in the promoter region of *CEBPA*. HDAC9 expression is downregulated during adipogenesis, resulting in its segregation from the USF1 complex and an increase in C/EBPα expression [105]. HDAC9 is also involved in high-fat diet (HFD)-associated adipogenesis. Upregulation of HDAC9 blocks adipogenic differentiation in mice chronically fed a high-fat diet, leading to the accumulation of poorly differentiated adipocytes and reduced lipocalin expression [106]. A recent study showed that HDAC9, which is expressed in mature adipocytes, plays an important role in the regulation of adipose tissue function and metabolic diseases in female mice, but not in male mice, with significant sex differences [107]. HDAC11 promotes adipogenic differentiation by regulating the expression of essential transcription factors and adipokines. Inhibition of *HDAC11* by siRNA results in reduced expression of perilipin, ADIPOQ, and PPARγ2, as well as reduced intracellular lipid droplet formation, thereby inhibiting adipogenic differentiation [108]. HDAC11 is also involved in the regulation of adipocyte phenotype [109].

#### Different SIRTs have facilitative or inhibitory effects at different stages of adipocyte differentiation

SIRT1 promotes or inhibits adipogenesis at different stages and is a key regulator of preadipocyte proliferation, clonal expansion, and adipocyte differentiation, targeting transcription factors, such as PGC-1α, v-Myc avian myelocytomatosis viral oncogene homolog (c-Myc), and NCoR1 [110]. SIRT1 inhibited adipogenesis by interacting with NCoR1 and SMRT, thereby inhibiting PPARγ activity [111]. Overexpression of SIRT1 deacetylates histone promoter-secreted frizzled-related protein 1 (sFRP1), sFRP2, and DACT1 and activates the WNT signaling pathway, thereby inhibiting adipogenic differentiation in MSCs [112]. CDK2-associated cullin 1 (CACUL1) is a SIRT1-interacting protein that binds to PPARγ through co-blocking nuclear receptor (CoRNR) box 2 and inhibits the transcriptional activity of PPARγ by reducing its acetylation at H3K9 [113]. SIRT1 also prolongs the cell cycle of pre-dividing adipocytes and inhibits the clonal expansion of differentiated adipocytes by inhibiting c-Myc [114]. Retinoic acid (RA) promotes adipocyte formation in mouse embryonic stem cells (mESCs), and activation of retinoic acid receptors (RARs) by RA plays an important role in the adipogenic differentiation of mESCs [115,116]. NCoR1 inhibits RAR signaling, expression, and acetylation, and its function is regulated by SIRT1. SIRT1 deficiency inhibited adipogenesis by increasing NCoR1 acetylation and downregulating RARα and RARβ expression [117]. FOXO1 is a known inhibitor of adipogenesis and is regulated by both acetylation and deacetylation. SIRT1 plays a critical role in regulating metabolic pathways by deacetylating FOXO1 in the nucleus [118]. In response to growth factor stimulation, SIRT1 is mainly distributed in the nucleus and binds directly to FOXO1, deacetylating FOXO1, and ultimately increasing its transcriptional activity [119]. In addition, SIRT1 promoted beige adipocyte differentiation. SIRT1 deacetylates PPARγ, which activates the binding of this transcription factor to PRDM16 to regulate the expression of beige adipocyte-specific genes, such as uncoupling protein 1 (*UCP1*), cell death-inducing DFFA-like effector A (*CIDEA*), and cytochrome C oxidase subunit 8B (*COX8B*) [120]. SIRT1 promotes beige adipocyte differentiation via the P53/P21 pathway [121]. SIRT2 can inhibit adipogenesis through mechanisms such as increased acetylation of FOXO1 and direct interaction with FOXO1. During the MCE of 3T3-L1 adipocytes, *SIRT2*-knockdown enhanced FOXO1 acetylation and promoted AKT-mediated phosphorylation, which led to subsequent FOXO1 nuclear elimination, whereas SIRT2 overexpression resulted in the opposite findings [122]. SIRT2 also promotes FOXO1 binding to PPARγ and subsequently inhibits its transcriptional activity to suppress adipogenesis [123]. SIRT3 promotes adipose tissue differentiation. Induction of SIRT3 by its activator honokiol (HNK) results in increased adipogenesis and triglyceride levels in 3T3-L1 preadipocytes, whereas treatment with the SIRT3 inhibitor 3-(1H-1,2,3-triazol-4-yl) pyridine (3-TYP) reduces adipogenesis and triglyceride levels. This indicates that SIRT3 promotes adipocyte differentiation [124]. SIRT3 is also associated with the phenotype of brown adipocyte differentiation and is required for the response of brown adipocytes to PGC-1α to promote full cryogenic brown adipocyte differentiation [125]. SIRT3 depletion reduced FOXO3a protein levels and impaired the ability of adipose tissue-derived human MSCs to differentiate into adipocytes, leading to adipocyte dysfunction and insulin resistance [126]. SIRT4 is localized to the mitochondria. *SIRT4*-knockdown impairs adipocyte differentiation. Comparison of overexpression of SIRT4 wild type and a catalytically inactivated SIRT4 H162Y mutant (SIRT4HY) showed that overexpression of SIRT4HY did not increase the accumulation of oil red O, suggesting that adipogenesis requires full SIRT4 catalytic activity. Overexpression of SIRT4 enhanced the expression of the adipogenic factors C/EBPα, CIDEA, ACSL1, and PPARα. During the early stages of adipogenesis, mitochondrial SIRT4 enhances branched-chain amino acid (BCAA) catabolism by activating methylcrotonyl-CoA carboxylase (MCCC). MCCC supports leucine oxidation by catalyzing the carboxylation of 3-methylcrotonyl-CoA to 3-methylglutaryl-CoA. SIRT4 expression is reduced in adipose tissue in many diabetic mouse models and is mostly associated with BCAA enzymes, suggesting a potential role for SIRT4 in adipose pathology by altering BCAA metabolism [127]. *SIRT4*-knockdown significantly suppressed the expression of marker genes that promote bovine adipocyte differentiation, suggesting that SIRT4 is closely associated with adipogenesis [128]. Treatment of 3T3-L1 preadipocytes with the SIRT5 inhibitor MC3482 resulted in brown adipocyte differentiation and increased the expression of two key regulators of the brown-specific adipogenic program, i.e., PPARγ and PRDM16, confirming that SIRT5 inhibition promoted brown adipogenesis [129]. In bovine preadipocytes, SIRT5 promoted brown adipogenesis by activating AMPK. MAPK inhibition prevents bovine preadipocyte differentiation, lipid synthesis, and lipid deposition in adipocytes, as has been demonstrated in obese mice [42]. SIRT6 inhibited preadipocyte differentiation and acted synergistically with SIRT5 to reduce lipid deposition in preadipocytes by activating the AMPKa pathway [130]. SIRT6-deficiency results in severe adipogenic defects and reduces expression of adipogenic markers, including PPARγ, C/EBPα, aP2, and lipocalin. SIRT6 promotes the expansion of mitotic clones during adipogenesis by inhibiting the expression of kinesin family member C (KIFC) and enhancing casein kinase 2 (CK2) activity [131]. SIRT7 promotes adipogenesis by inhibiting autocatalytic activation of SIRT1 [132]. SIRT7 binds to PPARγ2 and deacetylates PPARγ at the K382 residue. C3H10T1/2-derived adipocytes expressing PPARγ2K382Q (an acetylated K-mimic) accumulated much less fat than those expressing wild-type PPARγ2 or PPARγ2K382R (a non-acetylated K-mimic) [133]. In conclusion, SIRT1 positively or negatively regulates adipogenesis at different stages of adipogenic differentiation. Taken together, SIRT7 promotes adipogenesis by inhibiting the autocatalytic activation of SIRT1, but it can inhibit adipogenesis by deacetylating PPARγ2; SIRT2 inhibits adipogenesis by deacetylating FOXO1; SIRT3 and SIRT4 promote lipogenesis; whereas SIRT5 and SIRT6 inhibit preadipocyte differentiation and lipid deposition.

### The role of histone deacetylase inhibitors in adipocyte differentiation

HDACi regulate adipocyte differentiation by inhibiting HDACs. HDACi induce cell cycle arrest at the G1/S or G2/M transcription stage, leading to cell differentiation and apoptosis. HDACi are divided into four basic structural categories: short-chain fatty acids (valproic acid, sodium butyrate [NaB], phenylbutyrate), isohydroxamic acids (suberoylanilide hydroxamic acid [SAHA], TSA), cyclic peptides (romidepsin [FK228], apicidin [CAS 183,506–66–3]), and benzamide (e.g., mocinostat [MGCD103], dolma statin [4SC-202]). Some hydroxamic acid-derived compounds (SAHA, 4SC-201, and PXD-101) are pan-HDACis. Such pan-inhibitors have low specificity, and can therefore inhibit different classes of HDACs [134]. HDACi affects not only the histone DNA complex but also the acetylation state of non-histone proteins (STAT3 and P53 transcription factors). Adipocyte differentiation can be differentially regulated, depending on the type of HDACi used. n-butyrate was the first HDACi which was reported in 1977 [135]. Subsequently, the first natural oxime acid-based HDACi, TSA, was identified in 1990 [136]. TSA can bind to the zinc ionic bond at the bottom of HDAC tubulovesicular structure with its isohydroxamic acid ligands and inhibit the HDAC as the HDACi [137]. In adipose differentiation, TSA can reverse the decrease of p-AMPK expression level induced by DMI treatment and inhibit adipogenesis in 3T3-L1 preadipocytes by activating the AMPK pathway [45]. C646 inhibits the expression of p300 but increases the expression of TIP60 and PCAF, ultimately increasing the acetylation level of H3K9 and leading to a series of biological events such as reduced cell activity, cell cycle arrest, and apoptosis. Furthermore, C646 promotes the differentiation of goat adipose-derived stem cells into adipocytes and affects their ability to differentiate towards neural stem cells [138]. The expression of adipogenic marker genes such as *FAS, AP2, PPARG*, resistin, *CEBPG, ADD1*/*SREBP1C*, and diphtherin was inhibited by apicidin treatment. Apicidin treatment induces the differentiation of fully differentiated adipocytes, and apicitin treatment results in a reduction in Oil Red O-stained adipocytes along with a decrease in the expression levels of adipogenic marker genes [139]. In contrast, NaB stimulates adipogenic gene expression and adipocyte differentiation by inhibiting HDAC activity and tissue hyperacetylation [140]. Treatment of C3H10T1/2 cells with a SIRT1 inhibitor (nicotinamide) and RNA resulted in conditions that promoted adipogenesis and adipocyte differentiation, which were blocked by repeated treatment and SIRT1 overexpression [141].

### Adipocyte differentiation of cancer cells

#### CSCs can be induced to differentiate into adipocytes

CSCs can be induced to differentiate into post-mitotic cell types such as adipocytes. CSCs are a subpopulation of cancer cells with long-term self-renewal ability, proliferative capacity, and differentiation potential that drive tumorigenesis and initiate heterogeneous cancer formation. CSCs were first identified in hematopoietic malignancies as early as the 1870s and are now widely identified and characterized in hematopoietic and solid cancers [142], [143], [144]. CSCs proliferate due to the rapid production of daughter cells, resulting in tumor growth. At this time, they enter a quiescent state until they are stimulated again under favorable conditions. This allows CSCs to remain stable during tumor invasion and metastasis. This may account for the delayed recurrence of tumors at the primary site or elsewhere in the body, several years after the initial diagnosis. CSCs are highly resistant to conventional blood and radiation therapies owing to their ability to flexibly transition between states [145]. These cells survive conventional anti-cancer treatments and evade immune responses, resulting in treatment resistance, disease recurrence, and metastasis. CSCs are attractive targets for novel therapies that aim to eliminate and eradicate cancer cells. CSCs exhibit MSC properties. A popular research topic in recent years has been the differentiation of CSCs, such as inducing them to form adipocytes and other postmitotic cell types to reduce the invasive metastatic capacity of cancers [146,147]. Polyploid giant cancer cells (PGCCs) are a subpopulation of cancer cells with stem-cell properties [148]. Ovarian and breast cancer cell lines treated with high concentrations of cobalt chloride can produce PGCCs and daughter cells via asymmetric division, which can then be induced to differentiate into adipocytes by incubation in an adipocyte induction medium. These cells have been shown to have reduced invasive metastatic capacity [13].

#### Cancer cells undergoing epithelial-mesenchymal transition have the potential to differentiate into adipocytes

In addition to CSCs, cancer cells undergoing EMT can differentiate into adipocytes. EMT is a process in which epithelial cells transform into mesenchymal cells and gain the ability to invade and migrate. Mesenchymal–epithelial transition (MET) is a reverse transformation process of EMT, in which tumor cells undergoing EMT become motile mesenchymal cells and migrate, whereas migrated cells revert to epithelial cell morphology and undergo rapid proliferation and growth through MET [149]. EMT plays an important role in promoting cancer cell plasticity and allows cancer cells to evade chemotherapy and targeted therapy through dedifferentiation and signal transduction. Post-EMT cancer cells exhibit a high degree of plasticity. Many studies have exploited this plasticity to investigate treatment options by inducing the differentiation of cancer cells into benign cells after forced EMT [150,151]. Previous studies have mostly focused on the reduction of cancer cells from solid tumors to their original differentiated cell types. However, the benefit of this reversal is limited because of the inherent plasticity of the reversed cells and driver mutations that continue to activate oncogenic pathways, leading to malignant progression. Recent studies have shown that differentiation into post-mitotic cell types such as adipocytes can overcome oncogenic pathway activation and dynamic cellular plasticity. After in vitro induction with rosiglitazone and bone morphogenetic protein-2 (BMP-2), post-EMT breast cancer cells were transformed into adipocytes expressing various adipocyte markers and responding to isoproterenol and insulin in the same manner as adipocytes. Moreover, genes that induce proliferation were downregulated in differentiated adipocytes, whereas those that inhibit the cell cycle were upregulated. Differentiated adipocytes lose their aggressiveness and proliferative capacity. In one study, after nine days of induced differentiation, the differentiation medium of adipocytes was replaced with a normal medium, and the differentiated adipocytes maintained their adipocyte characteristics and did not revert to mesenchymal-like cancer cells, demonstrating the irreversibility of such induced differentiation. The researchers then used the PPARγ agonist rosiglitazone (an anti-diabetic drug) in combination with the MEK inhibitor trametinib in a mouse breast cancer model and achieved differentiation of the breast cancer cells into adipocytes after EMT [12].

#### Mechanisms of adipocyte differentiation of cancer cells

The key steps in adipocyte differentiation in tumors are the same as those that activate the transcription factor PPARγ in bone marrow mesenchymal stem cells. In the case of CSC of liposarcoma, for example, PPARγ can form a DNA-binding complex with the heterodimeric chaperone retinoid X receptor (RXRa) to regulate the transcription of adipocyte-specific genes [152,153]. All-trans retinoic acid is a potent inhibitor of adipocyte differentiation, and this action is mainly mediated by retinoic acid receptors [154]. PPARγ and the RXRa complex respond to a distinct retinoid signaling pathway by binding 9-cis-RA [155]. Terminal differentiation of primary human liposarcoma cells can be induced by treatment with thiazolidinediones or dRXR-specific retinoids [156]. When induced by dexamethasone, indomethacin, insulin, and 3-isobuty-1–1methylxanthine (IBMX), highly differentiated and dedifferentiated liposarcoma cells differentiate into adipocytes, which show upregulation of adipocyte markers, reduced proliferation, and typical adipocyte morphology [157].

In the EMT phenotype, the higher plasticity of cancer cells with mesenchymal properties is due to a mechanistic link and functional overlap between EMT and CSC phenotypes [158]. Transdifferentiation of cells into adipocytes has been initiated using TGF-β to induce EMT [12]. However, this growth factor plays an inhibitory role in adipocyte differentiation and inhibits adipose transdifferentiation of EMT-derived breast cancer cells by activating MEK/ERK signaling [159,160]. Thus, using an MEK inhibitor (trametinib) in combination with the adipogenesis-inducer rosiglitazone for adipogenic transdifferentiation therapy strongly promoted the direct spectrum transformation of these cancer cells and prevented their invasion and metastasis. Normally, the TGF-β signaling pathway, of which MEK is a key protein, inhibits the transformation of adipocytes. The activation of MEK–ERK signaling hinders differentiation potential and promotes the formation of adipocytes if MEK function is inhibited. Therefore, the combination of MEK inhibitors and PPARγ agonists induces adipogenesis, which may lead to the transdifferentiation of cancer cells into true adipocytes [12].

### The role of acetylation modifications in the induction of cancer cell differentiation

#### HDACs and HDACi are involved in regulating the adipogenesis of several types of cancer cells

Acetylation-modifying enzymes have also been reported to be involved in regulating the differentiation of CSCs and cancer cells undergoing EMT towards mesenchymal phenotype cells. HDAC SIRT1 and PPARγ1 converge to control lipid metabolism in vivo. In experiments on mice conducted in vivo, acetylation-deficient mutants of PPARγ1 showed reduced lipid synthesis in ErbB2-overexpressing breast carcinoma cells. The results suggested that the conserved lysine residues K154/K155 of PPARγ1 are acetylated and play an important role in lipid synthesis in ErbB2-positive breast cancer cells [161]. Different HDACi induce differentiation and increase sensitivity to chemotherapy in EMT-derived pancreatic cancer cells. Treatment with SAHA, an HDACi, induced the differentiation of mesenchymal invasive breast cancer cells into epithelial cells. SAHA treatment reduced the proliferation and induced the differentiation of these cells. SAHA inhibits cell proliferation and accumulation in a dose-dependent manner in the G1 and G2-M phases of the MCF-7 human breast cancer cell cycle and induces milk lipoglobulin, milk-lipid membrane globulin, and lipid droplets [162]. The effects of SIRT1/2 inhibitors or agonists on H446 cells were investigated in another study. SIRT1/2 activation promotes cancer cell differentiation, suggesting that SIRT1/2 is a novel target for treating cancer cells by inducing lipid differentiation [163]. TSA upregulates the expression of BM88/cell cycle exit and neural differentiation protein 1 (CEND1), which is derived from neural crest stem cells, in neuroblastoma, and induces cancer cells to exit the cell cycle and differentiate. TSA also induces neural differentiation and apoptosis in NCI-H446 cells [164].

#### Inhibition of p53 pathway acetylation suppresses adipocyte differentiation of cancer cells

P53 regulates cell survival and proliferation and responds to a variety of stress signals for abnormal cell growth and tumorigenesis. In addition to its role as a tumor suppressor, P53 is involved in the regulation of mesenchymal cell differentiation and can inhibit white adipocyte differentiation. The inhibitory effect of P53 on adipocyte differentiation depends on its transcriptional activity during mitotic clonal amplification. Thus, a possible mechanism linking clonal amplification to adipogenesis is the ability of P53 to inhibit adipogenesis [165]. Inhibition of white adipocyte differentiation by P53 is a general phenomenon observed in both mouse and human cell lines [166]. P53 inhibition during white adipocyte differentiation may also be achieved by regulating the expression of several genes, including the gene encoding P21 [167,168]. For example, mouse embryonic fibroblasts (MEF) lacking genes encoding P53 or P21 undergo spontaneous adipogenesis, whereas mice lacking genes encoding P21 and P27 develop adipose tissue hyperplasia [169]. MEFs isolated from *Tp53*-knockout mice differentiate into adipocytes. MEFs obtained from *Tp53-*knockout mice accumulated more lipid droplets and showed increased expression of adipogenic markers (PPARγ and C/EBPα) compared to wild-type MEFs [165]. Twist family BHLH transcription factor 2 (TWIST2) is also a target protein of P53, which is essential for the regulation of adipocyte differentiation in bone marrow MSCs. P53 deletion induces reactive oxygen species efflux from the mitochondria and downregulates TWIST2 expression. This loss of adipogenic potential can prevent lipogenic differentiation of bone marrow MSCs and lead to osteogenic differentiation of these cells. These results suggest that P53 is important not only for adipogenesis but also for maintaining MSC integrity [170]. Although P53 deletion affects terminal differentiation, it may lead to advanced expression in colonic epithelial cells, leading to tumor transformation. This suggests a complex and/or indirect interplaP53y between P53 and PPARγ [166].

Protein post-translational modifications (PTMs) are important links in the regulatory network of P53 function, where acetylation modifications not only regulate the overall transcriptional activity and protein stability of P53, but also the selectivity of P53 for the transcription of certain types of target genes under specific conditions [171]. Daughter cells of PGCCs (PDCs) differentiate into adipocytes [148]. A study found that p300 interacts with P53 and regulates the expression of acetylated PPARγ in HEY and MDA-MB-231 PDCs after adipogenic differentiation, and correlates with the *p53* genotype. In MDA-MB-231 PDCs with mutant *p53*, the activity of p300 was inhibited by using an acetylation inhibitor of P53, thereby reducing adipocyte differentiation [13].

### Potential clinical applications of adipocyte differentiation of cancer cells

Owing to their characteristics, CSCs are considered the root cause of tumorigenesis, spread, and recurrence, and may lead to the failure of existing therapies to sustainably eradicate malignant cancers. Differentiation therapy has been widely investigated as a promising therapeutic approach. Previous research on differentiation therapy has been devoted to restoring cancer cells to their original differentiated cell type, which has been found in vivo and in vitro, as well as in clinical studies to trigger the malignant progression of oncogenic pathways that may be persistently activated. In contrast, the transdifferentiation of cancer cells into post-mitotic cell types such as adipocytes can overcome the activation of oncogenic pathways and dynamic cellular plasticity. Therefore, targeted induction of CSC differentiation restrains tumor progression by inducing loss of the self-renewal capacity of CSCs and depleting the CSC pool and is a promising approach for clinical application as an adjunct to conventional radiotherapy for solid tumors [172]. Enhanced cancer cell plasticity can be exploited by inducing the transdifferentiation of different cell types. In recent years, the study of induced differentiation of cancer cells has advanced and new differentiation-inducing agents have been explored, opening up a new avenue for clinical cancer therapy.

Transdifferentiation of CSCs in melanoma has been extensively studied, and these CSC subpopulations form spheroids, which have been shown to promote tumor protein formation in mice in vivo assays [173]. Melanoma spheroids can be induced to differentiate into multiple cell lines such as melanocytes, adipocytes, chondrocytes, and osteoblastic cells using different inducers [174]. Transdifferentiation of melanoma CSCs can be induced by upregulation of PPARγ [175]. PPARγ agonists, such as rosiglitazone, have been extensively studied in the induction of differentiation in malignant tumors and induce cell transdifferentiation in various malignancies. Trabectedin is approximately 80% effective in metastatic mucinous liposarcomas; however, cases of drug resistance still exist. The use of pioglitazone, another PPARγ agonist, in combination with trabectedin is superior to the use of trabectedin alone in inducing adipocyte differentiation [176]. In breast cancer cells, the use of an MEK inhibitor (trametinib) in combination with rosiglitazone for adipogenic transdifferentiation therapy strongly promoted the direct lineage differentiation of these cancer cells. The PPARγ agonist mycophenolic acid can also reverse the malignancy of breast cancer cells by inducing their differentiation into adipocytes by activating PPARγ [177]. In chronic myelogenous leukemia and glioblastoma multiforme, PPARγ agonists synergistically induce transformed cell differentiation [178,179]. Transdifferentiation into post-mitotic cell types such as adipocytes potently prevents the invasion and metastasis of cancer cells, confirming the potential of transdifferentiation therapy that exploits the inherent cellular plasticity of cancer cells.

Acetylation modifications targeting PPARγ and P53 have also been investigated in cancer-induced differentiation, which occurred in the PGCC lines HEY and MDA-MB-231 PDCs that underwent induction of lipogenic differentiation, and in MDA-MB-231 PDCs with mutant P53, treatment with histone acetylase inhibitors reduced histone acetylation enzyme p300 activity, and the reduction of p300 activity inhibited the acetylation of P53, thereby reducing lipidogenic differentiation [13].

However, differentiation-inducing drugs may have adverse effects in clinical settings. For example, in the treatment of APL, retinoic acid (RA), which is used to induce differentiation, is thought to exert immune tolerance through many types of immune cells. The pathogenesis of acute APL is due to the PML-RARα complex blocking the differentiation of promyelocytes, and all-trans retinoic acid degrades the PML-RARα complex, disrupting this blockage and allowing promyelocytes to differentiate. The cellular growth that accompanies cellular differentiation in the treatment of AML with RA does not constitute a side effect. However, in the presence of malignant cells, RA-inducing factors can have both positive and negative effects–inducing malignant differentiation positively but also promoting malignant cell growth negatively. The latter of which should be strictly avoided in clinical therapy [180].

Therefore, further exploration is needed for induced differentiation therapy of cancer cells, and questions such as whether drug-microenvironment interactions affect efficacy and how to cross the solid cancer barrier need to be addressed. Future research on cancer cell differentiation will provide further support for clinical treatments.

## Conclusion

Adipocyte differentiation is a complex, multifactorial, and multistep process, and epigenetic mechanisms are fundamentally involved in its transcriptional regulation of adipocyte differentiation. After directed lineage from pluripotent stem cells, adipocytes undergo growth arrest and mitotic clonal expansion, a key program in which is the expansion of a transcription factor cascade, phosphorylation of the transcription factor C/EBPβ by MAP kinase and GSK-3β. Activated C/EBPβ then triggers the transcription of PPARγ and C/EBPα, which in turn synergistically activates the expression of genes whose expression generates the adipocyte phenotype. For key transcription factors, histone acetylation plays an important role in the regulation of transcriptional pathways. In this review, we summarized the role of acetylation modifications in adipocyte differentiation. We aimed to demonstrate how epigenetic remodeling regulates the activation or repression of genes that determine stem cell fate during differentiation.

The ability of CSCs to flexibly switch between quiescent and proliferative states allows them to become highly resistant to conventional anticancer therapies and to evade immune responses that can lead to drug resistance, disease recurrence, and metastasis. Therefore, novel therapies targeting CSCs are a breakthrough in the treatment of cancer. CSCs and cancer cells undergoing EMT have MSC properties and can be induced to differentiate into adipocytes. Cancer differentiation therapy aims to induce the differentiation of cancer cells into a benign cell phenotype after mitosis, reducing their ability to invade and metastasize, which can fundamentally eliminate the source of cancer invasion and metastasis resistance compared to traditional therapies. This process is influenced by the acetylation and deacetylation of histones, which are the major transcription factors involved in adipose-induced differentiation. However, many questions need to be addressed, such as how to perform in vivo induction in the clinic and whether there are multiple negative effects of inducing drugs in a malignant cancer environment. Further studies targeting the acetylation modification of the adipose differentiation pathway for malignancy therapy will provide new evidence and ideas for clinical treatment.

## Ethics approval and consent to participate

This review does not contain any studies with human or animal subjects performed by any authors.

## Availability of data and material

The original contributions presented in the study are included in the article, further inquiries can be directed to the corresponding author on reasonable request.

## Fundings

This work was supported by grants from the National Science Foundation of China (#82173283 and #82103088) and the Foundation of the committee on science and technology of Tianjin (#21JCZDJC00230 and 21JCYBJC00190). The funders had no roles in the design of the study, data collection, analysis, interpretation, or decision to write and publish the work.

## CRediT authorship contribution statement

**Xiaorui Wang:** Writing – original draft. **Na Li:** Writing – original draft. **Minying Zheng:** Writing – review & editing. **Yongjun Yu:** Conceptualization, Supervision. **Shiwu Zhang:** Investigation, Writing – original draft, Writing – review & editing.

## Declaration of Competing Interest

The authors declare that they have no known competing financial interests or personal relationships that could have appeared to influence the work reported in this paper.
